# Agreement between patients and surgeons on assessments of the cosmetic outcomes of Mohs micrographic surgery: Results of a single-center blinded prospective study

**DOI:** 10.1016/j.jdin.2021.07.004

**Published:** 2021-08-23

**Authors:** Karim Saleh, Åsa Ingvar, Johan Kappelin, Christina Persson, Katarina Lundqvist, Ingela Ahnlide, Bertil Persson

**Affiliations:** a Division of Dermatology and Venereology, Department of Clinical Sciences, Lund University, Skåne University Hospital, Lund, Sweden; b Department of Clinical Sciences, Helsingborg Hospital, Helsingborg, Sweden

*To the Editor:* Patient satisfaction with wound reconstruction following Mohs micrographic surgery (MMS) is crucial.^1^, ^2^, ^3^, ^4^, ^5^ Whether patients and their surgeons assess wound cosmetic outcomes similarly is still unknown. We aimed to examine whether patients and their Mohs surgeons agree with respect to their assessments of the cosmetic outcomes of MMS.

Following ethical approval, all patients who underwent MMS at Skåne University Hospital between 2010 and 2018 were assessed 6 months after MMS. No postsurgical scar interventions were performed prior to this. Almost all the MMS procedures were facial. At the 6-month assessment, the patients scored their cosmetic outcomes by marking a visual analog scale, ranging from the worst to the best cosmetic outcomes. The surgeons who performed the MMS scored the cosmetic outcomes similarly. The patients and surgeons were blinded to each other, but the patient scores were not collected anonymously. A single investigator registered all the scores by measuring marking distances in millimeter. Prior to data analysis, the authors agreed on the following arbitrary outcome categories: scores of 0-39 mm were categorized as “poor,” 40-79 mm as “acceptable,” and 80-100 mm as “excellent.” Inter-rater reliability was calculated using the Kappa-Fleiss coefficient. Differences in mean scores were calculated using an independent *t* test. Statistical significance was set at *P* < .05. The strength of the association between the mean scores and MMS variables was determined using an Eta coefficient test.

Three hundred forty-four patients were included in the study (Table I). Patient scores were registered for 313 (91%) patients, and scores for the Mohs surgeons were registered for 310 (90%) patients. Agreement between the surgeon and patient scores was illustrated using a Bland-Altman scatter plot (Fig 1). Most patients and surgeons scored the cosmetic outcomes as excellent. The inter-rater reliability of score category showed a fair agreement between the patients and the Mohs surgeons (Kappa-Fleiss coefficient: 0.24). There were no differences in the agreement levels between the patients and surgeons based on patient age or anatomic scar localization. The Mohs surgeons had a lower mean score than the patients, but this difference was statistically not significant (*P* = .07). The number of patients who scored their outcomes better than the Mohs surgeons was significantly higher than the number of patients who scored their outcomes worse than the surgeons (*P* = .04). Finally, there were no significant associations between the scores and sex, the number of stages of MMS, defect area, or reconstruction method.

**Table I:** Illustrating patient demographics and scores registered by patients and Mohs surgeons

Patients	Total n = 344	Women n = 223	Men n = 121
Age (y), mean ± SD (range)	66.9 ± 12.7 (24-94)	66.19 ± 13 (24-94)	68.1 ± 11.9 (34-90)
Mean defect area (mm^2^) ± SD	599.6 ± 647.9		
Mean n of Mohs stage ± SD	2.1 ± 0.8		
Cosmetic scores		Patients	Surgeons
n (%)		313 (91)	310 (90.1)
VAS category:			
Poor, n (%)		30 (8.7)	17 (4.9)
Acceptable, n (%)		109 (31.7)	142 (41.3)
Excellent, n (%)		174 (50.6)	151 (43.9)
Mean score ± SD		77.9 ± 22.7	74.9 ± 18.9
Reconstruction method, mean ± SD:			
Primary closure		83.8 ± 18.4	81.3 ± 15.3
FTSG		76.9 ± 23.9	69.6 ± 19.8
Advancement flap		74.9 ± 27.9	78.3 ± 21
Rotational flap		75 ± 23.9	81.3 ± 17.6
Transposition flap		78.1 ± 22.1	77.5 ± 16.2
Secondary intention		75.7 ± 20.7	74.8 ± 18.5
Combination flap		82.6 ± 16.6	73.4 ± 17

*FTSG*, Full-thickness skin grafting; *VAS*, visual analog scale.

**Fig 1:**
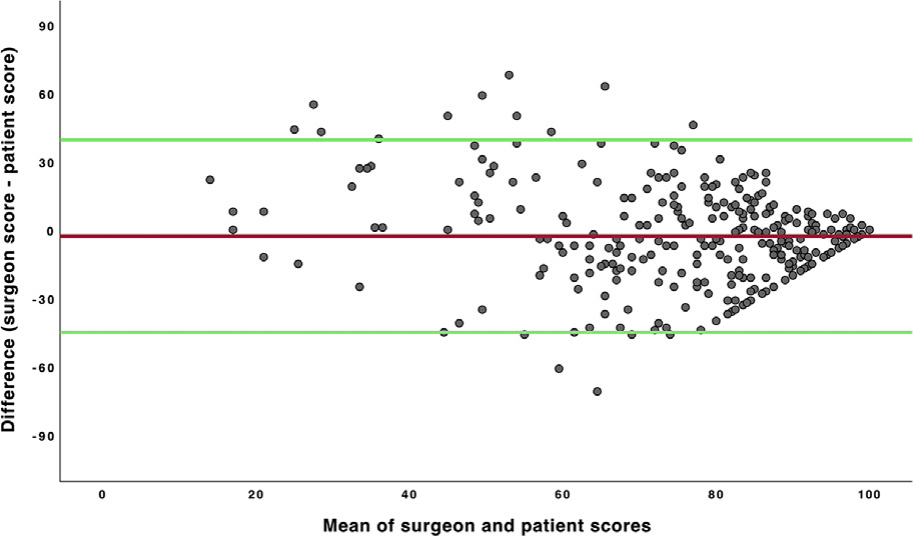
The Bland-Altman plot of cosmetic outcome scores in millimeter assessed by Mohs surgeons and patients at the follow-up visit after undergoing MMS. The y-axis illustrates score differences between the score of Mohs surgeons and patient score (score of Mohs surgeons − patient score), and the x-axis represents the means of the score of Mohs surgeons and patient score ([score of Mohs surgeons + patient score]/2). The *red* lines indicate the mean difference values, and the *green* lines indicate 95% reference intervals (the mean difference ± 1.96 × SD). *MMS*, Mohs micrographic surgery.

In summary, our study showed a fair inter-rater agreement when the patients' and surgeons' evaluations of the cosmetic outcomes of MMS were compared, which we believe is of value in managing reliable expectations after surgery. Another comforting finding was that most patients were satisfied with their results, and it was more common for a patient to rate his/her score better than a surgeon who performed MMS instead of the opposite. However, the Mohs surgeons, in general, tended to score cosmetic scores lower than the patients, but further studies are warranted to examine that in detail.

The study limitations included nonanonymous patient scores, a single-center study design, and the categorization of visual analog scale scores that have not been validated in previous studies.

## Conflicts of interest

None disclosed.
